# Ethyl 4-hy­droxy-2,6-diphenyl-1-(2-thio­morpholino­acet­yl)-1,2,5,6-tetra­hydro­pyridine-3-carboxyl­ate

**DOI:** 10.1107/S1600536810026413

**Published:** 2010-07-10

**Authors:** G. Aridoss, S. Sundaramoorthy, D. Velmurugan, K. S. Park, Y. T. Jeong

**Affiliations:** a Department of Image Science and Engineering, Pukyong National University, Busan 608-739, Republic of Korea; b Centre of Advanced Study in Crystallography and Biophysics, University of Madras, Guindy Campus, Chennai 600 025, India

## Abstract

In the title compound, C_26_H_30_N_2_O_4_S, the thio­morpholine ring adopts a chair conformation whereas the tetra­hydro­pyridine ring is in a half-chair conformation. The dihedral angle between the two phenyl rings is 33.3 (2)°. A strong intra­molecular O—H⋯O hydrogen bond generates an *S*(6) motif. In the crystal, mol­ecules are linked by inter­molecular C—H⋯O hydrogen bonds, generating a ribbon-like structure propagating along the *a* axis.

## Related literature

For general background to the biological activity of tetra­hydro­pyridine derivatives, see: Aridoss *et al.* (2008 ▶, 2010 ▶); Chow *et al.* (1968 ▶). For related structures, see: Subha Nandhini *et al.* (2003 ▶); Aridoss *et al.* (2009 ▶); Parkin *et al.* (2004 ▶). For ring conformational analysis, see: Cremer & Pople (1975 ▶); Nardelli (1983 ▶).
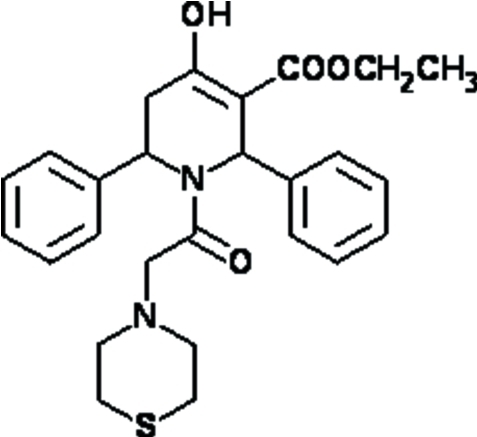


## Experimental

### 

#### Crystal data

C_26_H_30_N_2_O_4_S
*M* *_r_* = 466.58
Monoclinic, 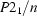

*a* = 10.9561 (6) Å
*b* = 9.5665 (6) Å
*c* = 22.9011 (12) Å
β = 93.575 (3)°
*V* = 2395.6 (2) Å^3^
*Z* = 4
Mo *K*α radiation
μ = 0.17 mm^−1^
*T* = 292 K
0.26 × 0.23 × 0.20 mm

#### Data collection

Bruker SMART APEXII area-detector diffractometer
Absorption correction: multi-scan (*SADABS*; Bruker, 2008 ▶) *T*_min_ = 0.957, *T*_max_ = 0.967
21473 measured reflections
5677 independent reflections
3669 reflections with *I* > 2σ(*I*)
*R*_int_ = 0.029

#### Refinement

*R*[*F* ^2^ > 2σ(*F* ^2^)] = 0.065
*wR*(*F* ^2^) = 0.217
*S* = 1.04
5677 reflections
299 parameters
1 restraint
H-atom parameters constrained
Δρ_max_ = 0.75 e Å^−3^
Δρ_min_ = −0.56 e Å^−3^

### 

Data collection: *APEX2* (Bruker, 2008 ▶); cell refinement: *SAINT* (Bruker, 2008 ▶); data reduction: *SAINT*; program(s) used to solve structure: *SHELXS97* (Sheldrick, 2008 ▶); program(s) used to refine structure: *SHELXL97* (Sheldrick, 2008 ▶); molecular graphics: *ORTEP-3* (Farrugia, 1997 ▶); software used to prepare material for publication: *SHELXL97* and *PLATON* (Spek, 2009 ▶).

## Supplementary Material

Crystal structure: contains datablocks global, I. DOI: 10.1107/S1600536810026413/ci5116sup1.cif

Structure factors: contains datablocks I. DOI: 10.1107/S1600536810026413/ci5116Isup2.hkl

Additional supplementary materials:  crystallographic information; 3D view; checkCIF report

## Figures and Tables

**Table 1:** Hydrogen-bond geometry (Å, °)

*D*—H⋯*A*	*D*—H	H⋯*A*	*D*⋯*A*	*D*—H⋯*A*
O1—H1*A*⋯O2	0.82	1.92	2.627 (3)	144
C2—H2*A*⋯O4^i^	0.97	2.46	3.306 (3)	145
C10—H10⋯O4^ii^	0.93	2.41	3.339 (4)	178

Symmetry codes: (i) 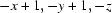; (ii) 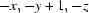.

**Fig. 1.**
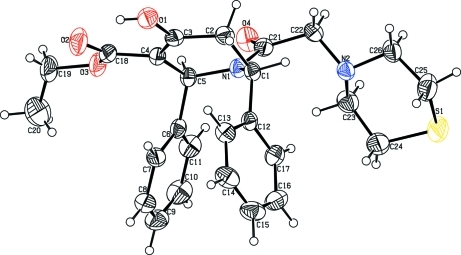
The molecular structure of the title compound, showing displacement ellipsoids drawn at the 30% probability level.

**Fig. 2.**
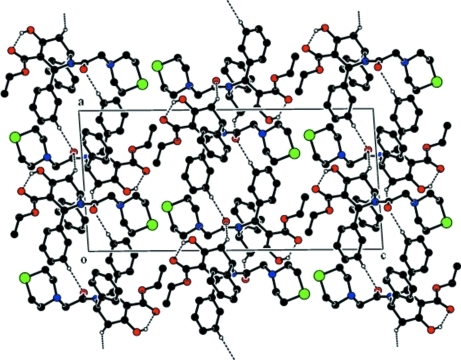
The crystal packing of the molecules viewed down the b axis. For clarity, H atoms which are not involved in hydrogen bonding are omitted.

## supplementary crystallographic information

### Comment

The asymmetric unit of *N*-chloroacetyl-3-carboxyethyl-2,6-diphenyl-4-hydroxy-Δ^3^-tetrahydropyridine obtained from the chloroacetylation of 3-carboxyethyl-2,6-diphenylpiperidin-4-one contains two crystallographically independent molecules wherein the two phenyl groups are oriented axially to avoid the steric repulsion (A^1,3^ strain; Chow *et al.*, 1968) with coplanar –NCOCH_2_ group besides adopting the non-chair conformation for the tetrahydropyridine ring (Aridoss *et al.*, 2008). However, it was confirmed by X-ray study that the two phenyl groups take up anti orientation with each other upon replacement of chlorine by morpholine system (Aridoss *et al.*, 2010). In order to study the orientation of phenyl groups and conformation of tetrahydropyridine ring system upon substitution of thiomorpholine instead of morpholine, the current study has been undertaken.

The sum of bond angles around atoms N1 (358.0 (2)°) and N2 (329.5 (2)°) of the tetrahydropyridine and the thiomorpholine rings in the molecule is in accordance with *sp*^2^ and *sp*^3^ hybridizations. The thiomorpholine ring adopts a chair conformation. The tetrahydropyridine ring adopts a half-chair conformation. The puckering parameters (Cremer & Pople, 1975) and the smallest displacement asymmetry parameters (Nardelli, 1983) for the thiomorpholine/tetrahydropyridine ring are q2 = 0.021 (3)/0.355 (2) Å, q3 = 0.631 (3)/-0.295 (2) Å; QT = 0.632 (3)/0.461 (2) Å and θ = 1.8 (3)/129.8 (3)°. The dihedral angle between the two phenyl ring is 33.4 (2)°. The thiomorpholine and tetrahydropyridine rings are connected by the ethanone. The ethyl acetate group shows an extended conformation [C18—O3—C19—C20 = 110.8 (6)°]. The molecular structure is stabilized by a strong O—H···O hydrogen bond, wherein, atom O1 acts as a donor to O2, generating an S(6) motif.

Atoms C2 and C10 act as donors to form hydrogen bonds with atom O4 as an aceptor. In the crystal structure, the molecules at (*x*,*y*,*z*) and (1 - *x*,1 - *y*,-*z*), (2 - *x*,1 - *y*,-*z*) are linked by C—H···O hydrogen bonds into a ribbon-like structure along the *a* axis; the ribbons contain *R_2_^2^*(12) and *R_2_^2^*(16) ring motifs.

### Experimental

To a mixture of thiomorpholine (1 equiv.) and dry K_2_CO_3_ (2 equiv.) in benzene, *N*-chloroacetyl-3-carboxyethyl-2,6-diphenylpiperidin-4-one (1 equiv.) in benzene was added slowly and refluxed until completion (Aridoss *et al.*, 2010). Through a typical work up procedure and purification, pure title compound was achieved, which on further crystallization in ethanol gave diffraction quality crystals.

### Refinement

H atoms were positioned geometrically (O–H = 0.82 Å and C–H = 0.93–0.98 Å) and allowed to ride on their parent atoms, with U_iso_(H) = 1.5*U*_eq_(C) for methyl H and 1.2 *U*_eq_(C) for other H atoms.

### Crystal data

**Table:**

C_26_H_30_N_2_O_4_S	*F*(000) = 992
*M**_r_* = 466.58	*D*_x_ = 1.294 Mg m^−^^3^
Monoclinic, *P*2_1_/*n*	Mo *K*α radiation, λ = 0.71073 Å
Hall symbol: -P 2yn	Cell parameters from 1201 reflections
*a* = 10.9561 (6) Å	θ = 1.8–28.2°
*b* = 9.5665 (6) Å	µ = 0.17 mm^−^^1^
*c* = 22.9011 (12) Å	*T* = 292 K
β = 93.575 (3)°	Block, colourless
*V* = 2395.6 (2) Å^3^	0.26 × 0.23 × 0.20 mm
*Z* = 4	

### Data collection

**Table:**

Bruker SMART APEXII area-detector diffractometer	5677 independent reflections
Radiation source: fine-focus sealed tube	3669 reflections with *I* > 2σ(*I*)
graphite	*R*_int_ = 0.029
ω and φ scans	θ_max_ = 28.2°, θ_min_ = 1.8°
Absorption correction: multi-scan (*SADABS*; Bruker, 2008)	*h* = −14→14
*T*_min_ = 0.957, *T*_max_ = 0.967	*k* = −11→12
21473 measured reflections	*l* = −29→29

### Refinement

**Table:**

Refinement on *F*^2^	Primary atom site location: structure-invariant direct methods
Least-squares matrix: full	Secondary atom site location: difference Fourier map
*R*[*F*^2^ > 2σ(*F*^2^)] = 0.065	Hydrogen site location: inferred from neighbouring sites
*wR*(*F*^2^) = 0.217	H-atom parameters constrained
*S* = 1.04	*w* = 1/[σ^2^(*F*_o_^2^) + (0.1095*P*)^2^ + 1.0086*P*] where *P* = (*F*_o_^2^ + 2*F*_c_^2^)/3
5677 reflections	(Δ/σ)_max_ = 0.001
299 parameters	Δρ_max_ = 0.75 e Å^−^^3^
1 restraint	Δρ_min_ = −0.56 e Å^−^^3^

### Special details

**Table:**

Geometry. All e.s.d.'s (except the e.s.d. in the dihedral angle between two l.s. planes) are estimated using the full covariance matrix. The cell e.s.d.'s are taken into account individually in the estimation of e.s.d.'s in distances, angles and torsion angles; correlations between e.s.d.'s in cell parameters are only used when they are defined by crystal symmetry. An approximate (isotropic) treatment of cell e.s.d.'s is used for estimating e.s.d.'s involving l.s. planes.
Refinement. Refinement of *F*^2^ against ALL reflections. The weighted *R*-factor *wR* and goodness of fit *S* are based on *F*^2^, conventional *R*-factors *R* are based on *F*, with *F* set to zero for negative *F*^2^. The threshold expression of *F*^2^ > σ(*F*^2^) is used only for calculating *R*-factors(gt) *etc*. and is not relevant to the choice of reflections for refinement. *R*-factors based on *F*^2^ are statistically about twice as large as those based on *F*, and *R*- factors based on ALL data will be even larger.

### Fractional atomic coordinates and isotropic or equivalent isotropic displacement parameters (Å^2^)

**Table:**

	*x*	*y*	*z*	*U*_iso_*/*U*_eq_	
C1	0.4059 (2)	0.1850 (2)	−0.01465 (10)	0.0453 (5)	
H1	0.4343	0.1666	0.0260	0.054*	
C2	0.5190 (2)	0.2068 (3)	−0.04884 (11)	0.0513 (6)	
H2A	0.5772	0.2644	−0.0260	0.062*	
H2B	0.5573	0.1171	−0.0552	0.062*	
C3	0.4893 (2)	0.2748 (3)	−0.10615 (10)	0.0490 (6)	
C4	0.3873 (2)	0.3516 (3)	−0.11724 (10)	0.0478 (6)	
C5	0.2932 (2)	0.3731 (2)	−0.07271 (10)	0.0444 (5)	
H5	0.2859	0.4745	−0.0678	0.053*	
C6	0.1649 (2)	0.3208 (3)	−0.09247 (10)	0.0459 (5)	
C7	0.1448 (2)	0.2216 (3)	−0.13558 (11)	0.0532 (6)	
H7	0.2113	0.1804	−0.1522	0.064*	
C8	0.0270 (3)	0.1820 (4)	−0.15461 (14)	0.0680 (8)	
H8	0.0150	0.1150	−0.1838	0.082*	
C9	−0.0707 (3)	0.2415 (4)	−0.13036 (17)	0.0796 (10)	
H9	−0.1497	0.2155	−0.1432	0.095*	
C10	−0.0529 (3)	0.3394 (4)	−0.08723 (18)	0.0805 (10)	
H10	−0.1198	0.3792	−0.0705	0.097*	
C11	0.0651 (3)	0.3799 (3)	−0.06812 (14)	0.0634 (7)	
H11	0.0766	0.4470	−0.0389	0.076*	
C12	0.3253 (2)	0.0626 (2)	−0.03482 (10)	0.0440 (5)	
C13	0.3550 (2)	−0.0269 (3)	−0.07933 (11)	0.0544 (6)	
H13	0.4231	−0.0085	−0.1004	0.065*	
C14	0.2841 (3)	−0.1436 (3)	−0.09264 (15)	0.0699 (8)	
H14	0.3051	−0.2032	−0.1225	0.084*	
C15	0.1833 (3)	−0.1722 (3)	−0.06235 (16)	0.0722 (9)	
H15	0.1365	−0.2514	−0.0713	0.087*	
C16	0.1517 (3)	−0.0838 (3)	−0.01875 (15)	0.0698 (8)	
H16	0.0827	−0.1023	0.0017	0.084*	
C17	0.2222 (2)	0.0330 (3)	−0.00502 (12)	0.0563 (6)	
H17	0.2000	0.0926	0.0246	0.068*	
C18	0.3700 (2)	0.4250 (3)	−0.17259 (12)	0.0608 (7)	
C19	0.2455 (3)	0.5885 (5)	−0.22699 (16)	0.0963 (13)	
H19A	0.3047	0.5682	−0.2556	0.116*	
H19B	0.2436	0.6888	−0.2209	0.116*	
C20	0.1253 (5)	0.5390 (10)	−0.2479 (3)	0.210 (4)	
H20A	0.1195	0.4404	−0.2409	0.315*	
H20B	0.1132	0.5568	−0.2891	0.315*	
H20C	0.0638	0.5871	−0.2276	0.315*	
C21	0.3374 (2)	0.4067 (3)	0.03114 (11)	0.0508 (6)	
C22	0.3935 (3)	0.3617 (3)	0.09078 (11)	0.0566 (6)	
H22A	0.4770	0.3320	0.0863	0.068*	
H22B	0.3963	0.4421	0.1167	0.068*	
C23	0.2018 (3)	0.2891 (4)	0.12692 (14)	0.0677 (8)	
H23A	0.1630	0.3190	0.0898	0.081*	
H23B	0.2017	0.3677	0.1536	0.081*	
C24	0.1283 (3)	0.1705 (5)	0.15118 (15)	0.0811 (10)	
H24A	0.1292	0.0915	0.1246	0.097*	
H24B	0.0440	0.2003	0.1531	0.097*	
C25	0.3410 (3)	0.0918 (5)	0.20431 (15)	0.0880 (11)	
H25A	0.3909	0.0721	0.2398	0.106*	
H25B	0.3453	0.0113	0.1788	0.106*	
C26	0.3931 (3)	0.2171 (4)	0.17450 (11)	0.0661 (8)	
H26A	0.3891	0.2977	0.2000	0.079*	
H26B	0.4785	0.1996	0.1682	0.079*	
N1	0.33686 (18)	0.3177 (2)	−0.01508 (8)	0.0449 (5)	
N2	0.32827 (18)	0.2491 (2)	0.11832 (8)	0.0508 (5)	
O1	0.57597 (16)	0.2550 (2)	−0.14448 (8)	0.0655 (5)	
H1A	0.5564	0.2960	−0.1751	0.098*	
O2	0.4311 (2)	0.4081 (3)	−0.21421 (9)	0.0931 (8)	
O3	0.27914 (19)	0.5182 (2)	−0.17266 (9)	0.0733 (6)	
O4	0.2948 (2)	0.5239 (2)	0.02621 (9)	0.0703 (6)	
S1	0.18591 (8)	0.11578 (13)	0.22245 (4)	0.0894 (4)	

### Atomic displacement parameters (Å^2^)

**Table:**

	*U*^11^	*U*^22^	*U*^33^	*U*^12^	*U*^13^	*U*^23^
C1	0.0494 (13)	0.0440 (13)	0.0416 (11)	0.0044 (10)	−0.0030 (9)	0.0038 (10)
C2	0.0460 (13)	0.0507 (14)	0.0563 (14)	0.0030 (11)	−0.0027 (11)	0.0048 (11)
C3	0.0409 (12)	0.0581 (15)	0.0481 (12)	−0.0064 (11)	0.0041 (10)	−0.0007 (11)
C4	0.0444 (12)	0.0556 (14)	0.0431 (12)	−0.0040 (11)	0.0004 (9)	0.0086 (11)
C5	0.0494 (13)	0.0414 (12)	0.0422 (11)	0.0047 (10)	0.0009 (9)	0.0064 (10)
C6	0.0476 (13)	0.0462 (13)	0.0440 (11)	0.0069 (10)	0.0036 (10)	0.0127 (10)
C7	0.0455 (13)	0.0633 (16)	0.0511 (13)	0.0012 (11)	0.0045 (10)	0.0004 (12)
C8	0.0566 (16)	0.079 (2)	0.0674 (17)	−0.0105 (15)	−0.0065 (13)	0.0080 (15)
C9	0.0430 (15)	0.096 (3)	0.098 (2)	−0.0052 (16)	−0.0040 (15)	0.027 (2)
C10	0.0493 (17)	0.090 (2)	0.104 (3)	0.0202 (16)	0.0206 (17)	0.014 (2)
C11	0.0561 (16)	0.0626 (17)	0.0726 (18)	0.0134 (13)	0.0131 (13)	0.0026 (14)
C12	0.0491 (13)	0.0402 (12)	0.0422 (11)	0.0059 (10)	−0.0014 (9)	0.0066 (10)
C13	0.0545 (14)	0.0551 (15)	0.0537 (13)	0.0040 (12)	0.0035 (11)	−0.0063 (12)
C14	0.0706 (19)	0.0600 (17)	0.0775 (19)	0.0073 (15)	−0.0081 (15)	−0.0180 (15)
C15	0.0669 (19)	0.0492 (16)	0.098 (2)	−0.0057 (14)	−0.0158 (17)	−0.0008 (16)
C16	0.0587 (17)	0.0653 (18)	0.086 (2)	−0.0083 (14)	0.0083 (15)	0.0139 (17)
C17	0.0602 (16)	0.0533 (15)	0.0562 (14)	0.0028 (12)	0.0096 (12)	0.0024 (12)
C18	0.0461 (14)	0.083 (2)	0.0528 (14)	−0.0113 (14)	−0.0002 (11)	0.0193 (14)
C19	0.090 (2)	0.122 (3)	0.074 (2)	−0.001 (2)	−0.0136 (18)	0.054 (2)
C20	0.161 (6)	0.285 (9)	0.170 (6)	−0.070 (6)	−0.097 (5)	0.132 (6)
C21	0.0545 (14)	0.0468 (14)	0.0512 (13)	−0.0051 (11)	0.0032 (11)	−0.0029 (11)
C22	0.0628 (16)	0.0607 (16)	0.0457 (13)	−0.0097 (13)	−0.0010 (11)	−0.0088 (12)
C23	0.0533 (15)	0.085 (2)	0.0649 (17)	0.0086 (15)	0.0013 (13)	−0.0027 (15)
C24	0.0572 (17)	0.115 (3)	0.0716 (19)	−0.0078 (18)	0.0067 (14)	0.003 (2)
C25	0.075 (2)	0.125 (3)	0.0650 (18)	0.014 (2)	0.0171 (16)	0.027 (2)
C26	0.0517 (15)	0.100 (2)	0.0464 (13)	0.0049 (15)	0.0036 (11)	0.0035 (14)
N1	0.0541 (11)	0.0394 (10)	0.0407 (9)	0.0032 (8)	−0.0006 (8)	0.0025 (8)
N2	0.0490 (11)	0.0620 (13)	0.0415 (10)	−0.0003 (10)	0.0031 (8)	−0.0024 (9)
O1	0.0481 (10)	0.0906 (15)	0.0588 (11)	0.0004 (10)	0.0102 (8)	0.0043 (10)
O2	0.0701 (14)	0.153 (2)	0.0575 (12)	0.0105 (15)	0.0187 (10)	0.0351 (14)
O3	0.0670 (12)	0.0914 (15)	0.0606 (11)	0.0040 (11)	−0.0022 (9)	0.0363 (11)
O4	0.0921 (15)	0.0517 (12)	0.0667 (12)	0.0117 (10)	0.0025 (11)	−0.0090 (9)
S1	0.0735 (6)	0.1382 (9)	0.0589 (5)	−0.0044 (5)	0.0220 (4)	0.0086 (5)

### Geometric parameters (Å, °)

**Table:**

C1—N1	1.477 (3)	C16—C17	1.383 (4)
C1—C2	1.521 (3)	C16—H16	0.93
C1—C12	1.521 (3)	C17—H17	0.93
C1—H1	0.98	C18—O2	1.209 (3)
C2—C3	1.483 (3)	C18—O3	1.336 (4)
C2—H2A	0.97	C19—O3	1.442 (3)
C2—H2B	0.97	C19—C20	1.452 (4)
C3—O1	1.346 (3)	C19—H19A	0.97
C3—C4	1.348 (3)	C19—H19B	0.97
C4—C18	1.451 (3)	C20—H20A	0.96
C4—C5	1.508 (3)	C20—H20B	0.96
C5—N1	1.474 (3)	C20—H20C	0.96
C5—C6	1.533 (3)	C21—O4	1.217 (3)
C5—H5	0.98	C21—N1	1.359 (3)
C6—C7	1.377 (4)	C21—C22	1.524 (4)
C6—C11	1.379 (4)	C22—N2	1.458 (3)
C7—C8	1.389 (4)	C22—H22A	0.97
C7—H7	0.93	C22—H22B	0.97
C8—C9	1.362 (5)	C23—N2	1.462 (3)
C8—H8	0.93	C23—C24	1.517 (5)
C9—C10	1.366 (5)	C23—H23A	0.97
C9—H9	0.93	C23—H23B	0.97
C10—C11	1.394 (5)	C24—S1	1.791 (4)
C10—H10	0.93	C24—H24A	0.97
C11—H11	0.93	C24—H24B	0.97
C12—C13	1.385 (3)	C25—C26	1.509 (5)
C12—C17	1.385 (4)	C25—S1	1.789 (3)
C13—C14	1.383 (4)	C25—H25A	0.97
C13—H13	0.93	C25—H25B	0.97
C14—C15	1.368 (5)	C26—N2	1.463 (3)
C14—H14	0.93	C26—H26A	0.97
C15—C16	1.370 (5)	C26—H26B	0.97
C15—H15	0.93	O1—H1A	0.82
			
N1—C1—C2	108.19 (19)	C12—C17—H17	119.5
N1—C1—C12	111.88 (19)	O2—C18—O3	122.5 (3)
C2—C1—C12	115.2 (2)	O2—C18—C4	125.1 (3)
N1—C1—H1	107.1	O3—C18—C4	112.4 (2)
C2—C1—H1	107.1	O3—C19—C20	108.1 (3)
C12—C1—H1	107.1	O3—C19—H19A	110.1
C3—C2—C1	111.97 (19)	C20—C19—H19A	110.1
C3—C2—H2A	109.2	O3—C19—H19B	110.1
C1—C2—H2A	109.2	C20—C19—H19B	110.1
C3—C2—H2B	109.2	H19A—C19—H19B	108.4
C1—C2—H2B	109.2	C19—C20—H20A	109.5
H2A—C2—H2B	107.9	C19—C20—H20B	109.5
O1—C3—C4	124.3 (2)	H20A—C20—H20B	109.5
O1—C3—C2	113.0 (2)	C19—C20—H20C	109.5
C4—C3—C2	122.7 (2)	H20A—C20—H20C	109.5
C3—C4—C18	119.3 (2)	H20B—C20—H20C	109.5
C3—C4—C5	122.7 (2)	O4—C21—N1	121.5 (2)
C18—C4—C5	117.8 (2)	O4—C21—C22	118.3 (2)
N1—C5—C4	111.03 (18)	N1—C21—C22	120.2 (2)
N1—C5—C6	112.77 (18)	N2—C22—C21	114.6 (2)
C4—C5—C6	114.10 (19)	N2—C22—H22A	108.6
N1—C5—H5	106.1	C21—C22—H22A	108.6
C4—C5—H5	106.1	N2—C22—H22B	108.6
C6—C5—H5	106.1	C21—C22—H22B	108.6
C7—C6—C11	118.5 (2)	H22A—C22—H22B	107.6
C7—C6—C5	122.6 (2)	N2—C23—C24	112.5 (3)
C11—C6—C5	118.8 (2)	N2—C23—H23A	109.1
C6—C7—C8	121.1 (3)	C24—C23—H23A	109.1
C6—C7—H7	119.4	N2—C23—H23B	109.1
C8—C7—H7	119.4	C24—C23—H23B	109.1
C9—C8—C7	119.8 (3)	H23A—C23—H23B	107.8
C9—C8—H8	120.1	C23—C24—S1	112.8 (2)
C7—C8—H8	120.1	C23—C24—H24A	109.0
C8—C9—C10	120.0 (3)	S1—C24—H24A	109.0
C8—C9—H9	120.0	C23—C24—H24B	109.0
C10—C9—H9	120.0	S1—C24—H24B	109.0
C9—C10—C11	120.4 (3)	H24A—C24—H24B	107.8
C9—C10—H10	119.8	C26—C25—S1	113.3 (3)
C11—C10—H10	119.8	C26—C25—H25A	108.9
C6—C11—C10	120.1 (3)	S1—C25—H25A	108.9
C6—C11—H11	119.9	C26—C25—H25B	108.9
C10—C11—H11	119.9	S1—C25—H25B	108.9
C13—C12—C17	118.2 (2)	H25A—C25—H25B	107.7
C13—C12—C1	122.8 (2)	N2—C26—C25	112.8 (3)
C17—C12—C1	118.9 (2)	N2—C26—H26A	109.0
C14—C13—C12	120.4 (3)	C25—C26—H26A	109.0
C14—C13—H13	119.8	N2—C26—H26B	109.0
C12—C13—H13	119.8	C25—C26—H26B	109.0
C15—C14—C13	120.7 (3)	H26A—C26—H26B	107.8
C15—C14—H14	119.7	C21—N1—C5	117.2 (2)
C13—C14—H14	119.7	C21—N1—C1	123.8 (2)
C14—C15—C16	119.7 (3)	C5—N1—C1	116.92 (18)
C14—C15—H15	120.2	C22—N2—C23	111.0 (2)
C16—C15—H15	120.2	C22—N2—C26	108.1 (2)
C15—C16—C17	120.1 (3)	C23—N2—C26	110.4 (2)
C15—C16—H16	119.9	C3—O1—H1A	109.5
C17—C16—H16	119.9	C18—O3—C19	117.6 (3)
C16—C17—C12	120.9 (3)	C25—S1—C24	96.42 (16)
C16—C17—H17	119.5		
			
N1—C1—C2—C3	−48.5 (3)	C13—C12—C17—C16	1.0 (4)
C12—C1—C2—C3	77.5 (3)	C1—C12—C17—C16	−174.8 (2)
C1—C2—C3—O1	−159.7 (2)	C3—C4—C18—O2	11.3 (5)
C1—C2—C3—C4	22.2 (3)	C5—C4—C18—O2	−172.8 (3)
O1—C3—C4—C18	−3.2 (4)	C3—C4—C18—O3	−167.2 (2)
C2—C3—C4—C18	174.5 (2)	C5—C4—C18—O3	8.8 (3)
O1—C3—C4—C5	−179.0 (2)	O4—C21—C22—N2	114.2 (3)
C2—C3—C4—C5	−1.2 (4)	N1—C21—C22—N2	−66.5 (3)
C3—C4—C5—N1	8.2 (3)	N2—C23—C24—S1	63.1 (3)
C18—C4—C5—N1	−167.6 (2)	S1—C25—C26—N2	−62.4 (3)
C3—C4—C5—C6	−120.6 (3)	O4—C21—N1—C5	5.6 (4)
C18—C4—C5—C6	63.6 (3)	C22—C21—N1—C5	−173.6 (2)
N1—C5—C6—C7	−105.9 (3)	O4—C21—N1—C1	168.7 (2)
C4—C5—C6—C7	22.0 (3)	C22—C21—N1—C1	−10.6 (4)
N1—C5—C6—C11	77.1 (3)	C4—C5—N1—C21	125.2 (2)
C4—C5—C6—C11	−155.0 (2)	C6—C5—N1—C21	−105.3 (2)
C11—C6—C7—C8	0.4 (4)	C4—C5—N1—C1	−39.1 (3)
C5—C6—C7—C8	−176.5 (2)	C6—C5—N1—C1	90.4 (2)
C6—C7—C8—C9	−0.2 (4)	C2—C1—N1—C21	−103.0 (2)
C7—C8—C9—C10	−0.3 (5)	C12—C1—N1—C21	129.1 (2)
C8—C9—C10—C11	0.6 (5)	C2—C1—N1—C5	60.1 (3)
C7—C6—C11—C10	−0.2 (4)	C12—C1—N1—C5	−67.8 (2)
C5—C6—C11—C10	176.9 (3)	C21—C22—N2—C23	−59.2 (3)
C9—C10—C11—C6	−0.3 (5)	C21—C22—N2—C26	179.6 (2)
N1—C1—C12—C13	126.8 (2)	C24—C23—N2—C22	175.6 (2)
C2—C1—C12—C13	2.8 (3)	C24—C23—N2—C26	−64.6 (3)
N1—C1—C12—C17	−57.6 (3)	C25—C26—N2—C22	−174.2 (2)
C2—C1—C12—C17	178.3 (2)	C25—C26—N2—C23	64.2 (3)
C17—C12—C13—C14	−1.1 (4)	O2—C18—O3—C19	7.2 (4)
C1—C12—C13—C14	174.5 (2)	C4—C18—O3—C19	−174.3 (3)
C12—C13—C14—C15	0.3 (4)	C20—C19—O3—C18	110.8 (6)
C13—C14—C15—C16	0.6 (5)	C26—C25—S1—C24	51.6 (3)
C14—C15—C16—C17	−0.7 (5)	C23—C24—S1—C25	−51.8 (3)
C15—C16—C17—C12	−0.1 (4)		

### Hydrogen-bond geometry (Å, °)

**Table:**

*D*—H···*A*	*D*—H	H···*A*	*D*···*A*	*D*—H···*A*
O1—H1A···O2	0.82	1.92	2.627 (3)	144
C2—H2A···O4^i^	0.97	2.46	3.306 (3)	145
C10—H10···O4^ii^	0.93	2.41	3.339 (4)	178

Symmetry codes: (i) −*x*+1, −*y*+1, −*z*; (ii) −*x*, −*y*+1, −*z*.
